# Uncovering by Atomic Force Microscopy of an original circular structure at the yeast cell surface in response to heat shock

**DOI:** 10.1186/1741-7007-12-6

**Published:** 2014-01-27

**Authors:** Flavien Pillet, Stéphane Lemonier, Marion Schiavone, Cécile Formosa, Hélène Martin-Yken, Jean Marie Francois, Etienne Dague

**Affiliations:** 1 CNRS, LAAS, 7 avenue du colonel Roche, F-31077 Toulouse, France; 2 Université de Toulouse, UPS, INSA, INP, ISAE, LAAS, F-31077 Toulouse, France; 3 CNRS, ITAV-USR 3505, F31106 Toulouse, France; 4 Université de Toulouse, INSA, UPS, INP, 135 avenue de Rangueil, F-31077 Toulouse, France; 5 INRA, UMR792 Ingénierie des Systèmes Biologiques et des Procédés, F-31077 Toulouse, France; 6 CNRS, UMR5504, F-31400 Toulouse, France; 7 CNRS, UMR 7565, SRSMC, Vandoeuvre-lès-Nancy, France; 8 Université de Lorraine, UMR 7565, Faculté de Pharmacie, Nancy, France

**Keywords:** Atomic Force Microscopy (AFM), *Saccharomyces cerevisiae*, Heat-shock, Cell wall, Chitin, Budding

## Abstract

**Background:**

Atomic Force Microscopy (AFM) is a polyvalent tool that allows biological and mechanical studies of full living microorganisms, and therefore the comprehension of molecular mechanisms at the nanoscale level. By combining AFM with genetical and biochemical methods, we explored the biophysical response of the yeast *Saccharomyces cerevisiae* to a temperature stress from 30°C to 42°C during 1 h.

**Results:**

We report for the first time the formation of an unprecedented circular structure at the cell surface that takes its origin at a single punctuate source and propagates in a concentric manner to reach a diameter of 2–3 μm at least, thus significantly greater than a bud scar. Concomitantly, the cell wall stiffness determined by the Young’s Modulus of heat stressed cells increased two fold with a concurrent increase of chitin. This heat-induced circular structure was not found either in *wsc1Δ* or *bck1Δ* mutants that are defective in the CWI signaling pathway, nor in *chs1Δ*, *chs3Δ* and *bni1Δ* mutant cells, reported to be deficient in the proper budding process. It was also abolished in the presence of latrunculin A, a toxin known to destabilize actin cytoskeleton.

**Conclusions:**

Our results suggest that this singular morphological event occurring at the cell surface is due to a dysfunction in the budding machinery caused by the heat shock and that this phenomenon is under the control of the CWI pathway.

## Background

The yeast *Saccharomyces cerevisiae* is a unicellular eukaryotic microorganism surrounded by a 100–120 nm thick cell wall [1]. The fungal cell wall is an essential structure that maintains cell shape and cell integrity, ensures resistance to internal turgor pressure and thereby prevents cell lysis [2]. The cell wall of *Saccharomyces cerevisiae*, which represents 10 - 25% of the cell dry mass according to the culture and process conditions [3], consists of three types of polymers that are interconnected to produce a modular complex structure [4]. The inner layer of the cell wall is composed of a β-1,3-glucan network (80 - 90% of the total β-glucan) branched with chitin (1–2% of the cell wall). Together, they form a structure that is largely responsible for the mechanical strength of the whole cell wall [5,6]. In addition, β-1,6-linked glucans (8 - 18% of total β-glucans) are branched on the β-1,3-glucan network, and also linked to the mannoproteins that compose the outer layer [7,8]. The yeast cell wall is a dynamic structure, the molecular architecture of which is continuously remodeled during morphogenetic processes and growth [9]. It also undergoes remodeling in response to environmental stresses, such as ethanol and oxidative stress [10,11], thermal and osmotic stress [12-14], and in response to antifungal drugs such as allicin or caspofungin [15,16]. These remodeling processes are organized by a “cell wall rescue-mechanism” that relies on a combination of several signaling pathways, with a major role played by the PKC1-dependent cell wall integrity (CWI) pathway (reviewed in [9,17]). Important biochemical modifications identified so far during stresses were i) massive deposition of chitin that takes place in the lateral walls of both the mother cells and the growing buds, ii) an increased cross-linkage between chitin and β-1,3-glucan and iii) the appearance of novel linkages between cell wall proteins and chitin through β-1,6-glucan [18,19]. Altogether, these cell wall repair mechanisms have been considered as a mean to combat cell wall weakening caused by these stresses [4,20]. However, a direct visualization of the topography and nanomechanical changes associated to these biochemical and molecular changes induced by stresses was still missing to better understand the cell wall biogenesis and remodeling mechanism. The remarkable development of the Atomic Force Microscopy (AFM) technology, combined with genetical and molecular tools, is therefore powerful to fulfil this gap and investigate the dynamics of microbial cell surfaces in response to external cues [21,22].

In this study, we have investigated the effects of heat shock on the nanomechanical properties of the yeast cell wall. We chose this stress condition because of the large body of data available on the heat shock response in the yeast *Saccharomyces cerevisiae* (reviewed in [23]). In brief, this response is characterized at the genome level by an intense program of changes in gene expression leading to repression of protein biosynthetic machinery and the induction of a battery of genes encoding heat shock proteins (HSPs). The main metabolic and physiological changes reported in response to heat stress are an accumulation of trehalose and an inhibition of glycolysis [24,25], associated with a transient arrest of cell division. Heat shock also triggers the activation of the CWI pathway, resulting in a global transcriptomic change including the overexpression of genes encoding cell wall remodeling enzymes [26]. Although AFM analysis of temperature stress on yeast cells has been previously addressed by Adya *et al*. [27], we have revisited this stress because of two major technical concerns in the study reported by the latter authors. Firstly, the immobilization procedure they used could likely alter the cell viability and integrity since yeast cells were immobilized on glass slides by air-drying for more than 5 hr. Secondly, the stress was carried out at temperature ranging from 50 to 90°C, which is incompatible with yeast life and irrelevant in a biotechnological viewpoint.

Using a recent immobilization method that ensures the viability and integrity of the yeast cells [28], we showed that a temperature shift from 30 to 42°C induced the singular formation of circular rings that initiate at a single point on the yeast cell surface and expanded in a concentric manner to reach a diameter of 2 to 3 μm after 1 h of incubation. Appearance of this circular structure was accompanied by a twofold increase of chitin and by a raise of the cell wall stiffness. Furthermore, we showed that the formation of this unique circular structure was dependent on the budding process and was regulated by the CWI pathway.

## Results

### Heat shock induces the formation of a circular structure at the yeast cell surface

**Figure 1:**
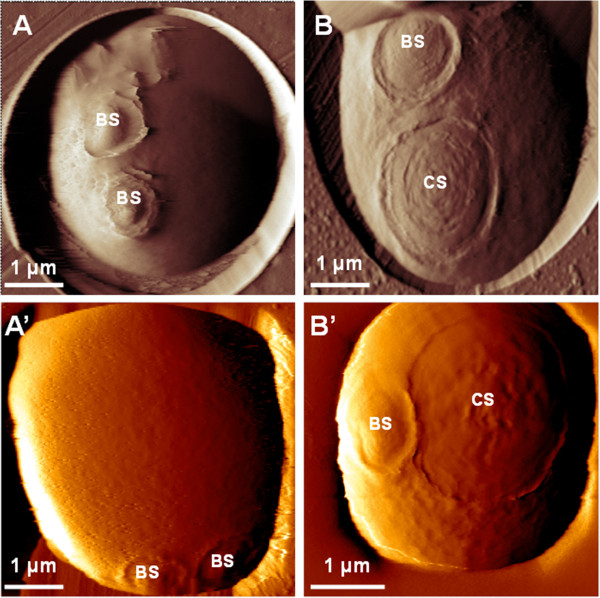
**Heat-shock exposition of yeast cells leads to the formation of an unexpected circular structure.** AFM deflection images of surface topology of a living yeast cell at 30°C (unstressed) **(A, A’)** or exposed to heat shock during 1 h at 42°C (heat-shocked) **(B, B’)**. Yeast cells were trapped in polycarbonate porous membrane (top panel) or within the patterns of a PDMS stamp (back panel). Bud scar (BS) and circular structure (CS) are indicated on AFM images.

To explore the heat shock effects on the yeast cell surface by AFM, a culture sample from exponentially growing yeast cells on YPD cultivated at 30°C was shifted at 42°C for 1 h. Both unstressed and heat shocked cells were then trapped in polycarbonate porous membrane (Figure 1, top panel) or immobilized in holes of a PDMS stamp (Figure 1; lower panel). The presence of two typical bud scars on the unstressed yeast cell was clearly identified on AFM deflection images (Figure 1A & A’). In contrast, the heat-shocked yeast cell presented beside a bud scar, a circular structure (CS) that had a size larger than the bud scar on its cell surface. This CS was not an epiphenomenon since it was observed over 20–25 individual heat shocked cells analyzed from three independent experiments. In addition, the formation of this unique CS was time dependent, since small concentric rings started to be observed after 20 min incubation at 42°C, and their number and size increased with time to finally covered the whole observable cell surface after 2 hr (data not shown). Also, we always observed only one CS per cell, although it could not be excluded that another circular structure was formed underside, since this side of the cells was not accessible to AFM study. This singular event was clearly associated with the heat shock response as witnessed by a rapid and huge accumulation of trehalose (Additional file 1: Figure S1), a key marker of the response of yeast to a thermal stress [24,29]. Also, the viability of heat shocked yeast cells after 1 h of treatment at 42°C was more than 99% as evaluated by methylene blue staining method (Additional file 2: Table S1).

### Ultrastructure of the cell surface CS using high resolution AFM imaging

**Figure 2:**
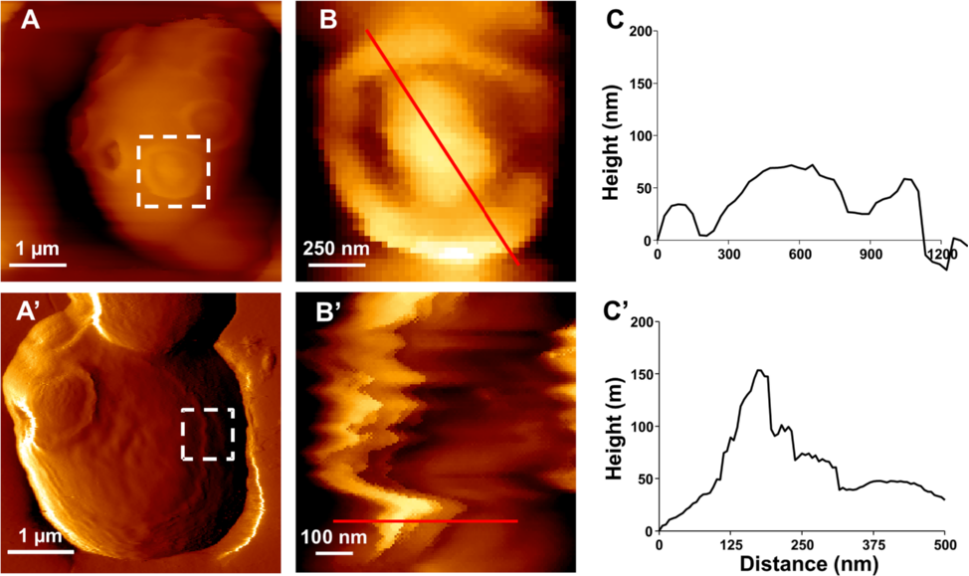
**Morphological differences between a bud scar (BS) and circular structure (CS) at the cell surface.** In **(A, A’)**, AFM deflection image of an unstressed and of a heat-shocked yeast cell after 1 hr at 42°C at a z range of 2.5 μm. The white dotted squares indicated AFM height image analysis for the BS **(A)** and for part of the CS **(A’)**. In **(B & B’)** are zoomed height images of these squares area (at z range of 200 nm). In **(C & C’)** are cross sections taken across the red lines, respectively in **B** and **B’** respectively.

**Figure 3:**
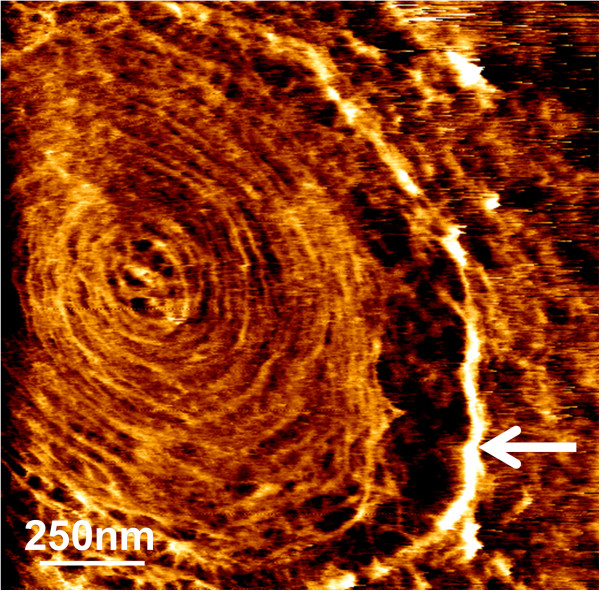
**Exploring the ultrastructure of a CS by AFM.** High-resolution deflection image shows a succession of concentric rings, followed by 1 major ring (white arrow).

To show that bud scar and CS were morphologically different, we carried out a detailed analysis of AFM height images on unstressed and heat shocked cells. A first difference was in the diameter of the two features, which was around 1 μm maximum for the bud scar but exceeded 2.5 μm for the cell surface CS (Figure 2). Also, the cross section taken on the AFM height image of the unstressed yeast cell confirmed the typical convex structure of the bud scar (Figure 2B & C), followed by a depression and terminated by an apparent rigid ring which corresponds to a local accumulation of chitin [30]. In contrast, the cell surface CS identified on the heat shocked cells showed a different morphology, being relatively smooth inside the structure and terminated by a sharp ring. At a higher resolution, the AFM deflection image allowed identifying a succession of circular rings that originated from a single point and expanded in a concentric manner to end up by one last sharp ring (Figure 3).

### Heat shock increases the yeast cell wall stiffness

**Figure 4:**
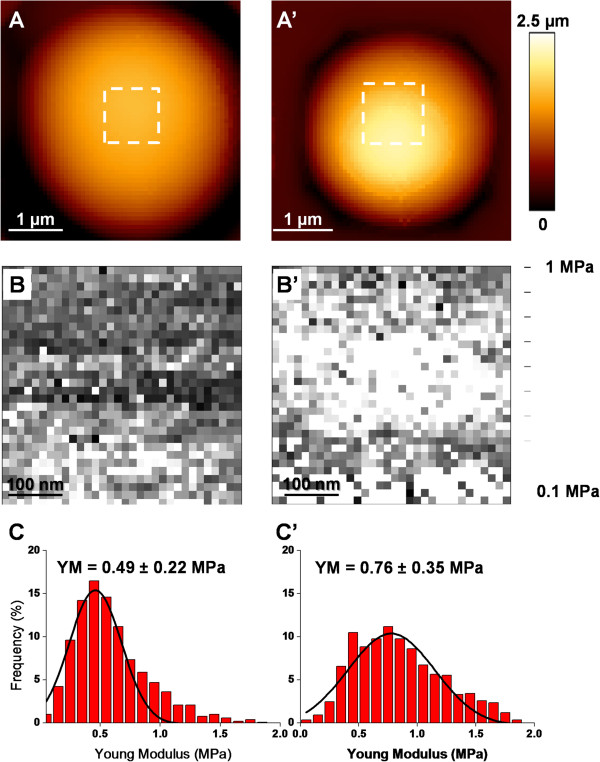
**Yeast stiffness is increased by heat-shock at 42 °C.** Young’s Modulus (YM) determinations on an unstressed **(A–C)** and a heat-shocked cells **(A’-C’)**. The white squares showed in the height images, (z range = 2 μm) **(A, A’),** indicate the localization of the elasticity maps shown in **(B, B’)**. Histograms of the YM distributions **(C, C’)** associated with the elasticity maps. YM medians were calculated by fitting a Gauss model (indicated by the black curves).

**Figure 5:**
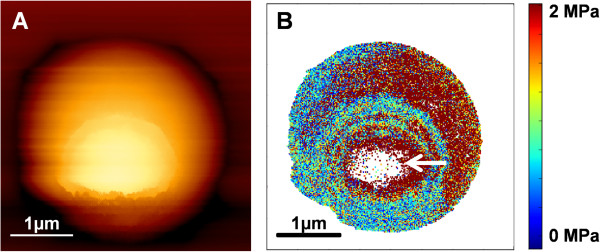
**Stiffness map of a heat-shocked yeast cell.** Height image (z range of 2.5 μm) **(A)**, with the corresponding elasticity map in quantitative mode, **(B)** at the z range of 2 MPa. A higher young modulus was characterized in the central part of CS (white arrow).

Quantitative data on the effects of heat shock were obtained by scanning a given area of the cell surface with the AFM tip. To this end, we choose an area on the cell that was elsewhere from bud and CS. Thousands of Force Volume (FV) measurements were recorded, translated into pixel units to yield an elasticity map from which Young’s Modulus (YM) values (expression of cell wall stiffness) could be calculated (Figure 4A & B). Qualitatively, the elasticity map of an unstressed yeast cell was homogeneous, while for the heat shocked cells, there was clearly a central region on the chosen area exhibiting higher pixel intensities, suggesting a difference in the elasticity or stiffness between the unstressed and the heat shocked cells. The YM values were extracted from all the force curves (*e.g*. 19443 FV curves from 19 unstressed cells, and 15307 FV curves from 15 heat-shocked yeast cells) and expressed as histograms that followed a Gauss distribution (Figure 4C and C’). The median values of the Gauss model fitting curve were used to determine YM from unstressed and heat-shocked cells. An unpaired *t*-test applied on the obtained YMs data (Additional file 3: Figure S2) allowed concluding that the YM from heat shocked was statistically twofold higher than that of unstressed yeast cells (*p* value < 0.0001). The same methodology was used to evaluate the YM at the CS vicinity of the heat shocked cells. As shown in Figure 5, the YM was even higher at the CS, reaching more than 2 MPa inside this structure. Taking into account that cell wall stiffness is generally correlated with changes in chitin level, this finding raised the question whether this increase of stiffness at the CS is linked to increase of chitin or to some other cell wall remodeling events.

### Chitin content in cell wall and link with cell wall stiffness

**Figure 6:**
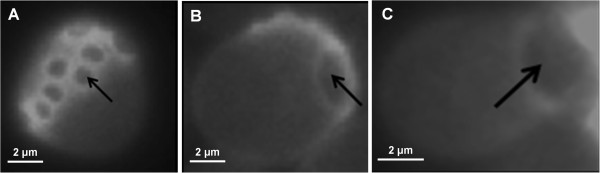
**Fluorescence images of calcofluor white stained yeast cells.** In **(A),** BY4741 cells cultivated at 30 °C showing bud scars. **(B & C),** BY4741 cells after 1 h of heat shock showing the circular structure **B** and **C**.

The formation of a cell surface CS and the increased stiffness suggested that the biochemical composition of the cell wall could have been modified in response to heat shock. To explore this hypothesis, we performed biochemical measurements of carbohydrate composition of the cell wall. As reported in Table 1, levels of β-glucan and mannans were not different between unstressed yeast cells and cells incubated at 42°C for 1 h. In contrast, the heat shock treatment clearly induced a 45% increase in the chitin content (from 46.2 μg/mg in unstressed cells to 68.3 μg/mg in cells after 1 h incubation at 42°C). To verify that this increase of chitin was preferentially associated with the formation of the CS, we visualized this polymer after staining it with calcofluor white (CFW). As expected, the presence of bud scars with a diameter around 1 μm was clearly visible on a yeast cell cultivated at 30°C (Figure 6A). However, it was interesting to notice that a ring of chitin with a diameter above 2 μm roughly co-localized with the CS in a heat shocked cell for 1 hr at 42°C (Figure 6B & C). Taken together, these result suggested that the increase of cells stiffness in response to heat shock may be linked to chitin levels.

**Table 1:** The chitin content in cell wall is increased upon heat-shock

	**Chitin**	**β-glucans**	**Mannans**
30°C	46.2 ± 9.5	440 ± 115	293 ± 24
42°C	68.3 ± 3.3	408 ± 99	301 ± 17

Carbohydrate composition of cell wall from unstressed (30°C) and heat-shocked cells during 1 h at 42°C were determined by acid hydrolysis for β-glucans and mannans and by enzymatic digestion for chitin. Values reported in μg per mg of dry cell wall are the mean ± SD of 3 biological independent experiments technically repeated 2 times.

### The formation of the cellular surface CS is dependent on the budding process

The finding that this singular CS was found on about 40% of the heat-shocked cells, showing some morphological signs of a bud, and produced at the vicinity of a previous bud, raised the hypothesis that this structure might be dependent upon the budding machinery system. The process of budding has been thoroughly investigated at the biological, genetic and molecular levels, and showed the implication of many genes and many structural and regulatory networks that encompass cell polarity, cytoskeleton, secretory pathway, cell signaling, etc. [31]. To provide a first biological evidence that the CS formation is dependent on the budding process, we used latrunculin A (LatA), a toxin known to destabilize the actin cytoskeleton [32] that is implicated in the budding process. Exponentially growing cells were subjected to a heat shock at 42°C for 1 hr in the presence of 200 μM LatA. On a sampling of 10 independent yeast cells, we were unable to observe any CS at the cell surface, as compared to results with heat-shocked cells not treated with LatA (Additional file 4: Figure S3A). Furthermore, in the absence of heat shock, the perturbation of the actin cytoskeleton by LatA did not lead to the formation of CS (Additional file 4: Figure S3BC). To get additional biological evidence that the CS involves the budding process, we performed heat shock experiments with exponentially growing yeast cells that were incubated in a nitrogen-depleted medium for 72 hr. This condition results in growth arrest in G1 phase of the cell cycle with virtually all the cells unbudded [33] They were then subjected to heat shock at 42°C for one hour and AFM analysis was carried out on 10 starved cells before and 1 hr after heat-shock. In none of the heat-shocked cells, could we find any CS at the cell surface (Additional file 5: Figure S4). This result can be taken as indirect evidence that CS is depending on the budding process, because of the inability of the nitrogen-starved yeast cells to bud both at 30 and 42°C. Thus, the circular structure only forms during active cell growth and this cannot be separated from a cell-cycle phase specific defect.

At the genetic level, we addressed this question using targeted mutants such as *chs3Δ* that is defective in chitin ring formation during bud emergence [34], *chs1Δ* since the loss of this gene impairs septum reparation during cytokinesis [35] as well as a mutant deleted for *BNI1* because this gene encodes a formin protein that is required for the proper initiation of bud growth and the proper shape of vegetative buds through formation of actin cables [36]. High resolution AFM imaging carried out on 15 cells from 3 independent experiments did not reveal any formation of singular cell surface CS in these different mutants after a heat shock at 42°C for 1 h (Additional file 6: Figure S5).

### The formation of the cellular surface CS is regulated by CWI pathway

Heat shock is known to activate the CWI pathway, and the surface sensor Wsc1 is one of the sensors that detect and transmit this cell wall stress to the signaling cascade [37]. To evaluate whether the formation of the cell surface CS was under the control of the CWI signaling and whether Wsc1 could be implicated in this response, both *bck1Δ* mutant defective in the MAP kinase of the CWI pathway [38] and *wsc1Δ* mutant cells were analyzed by AFM before and after 1 hr heat shock at 42°C. As compared to the wild type cells, which under this heat stress condition exhibited a large cell surface CS, neither the *bck1Δ* nor *wsc1Δ* cells imaged by AFM presented this singular structure (Additional file 7: Figure S6). The failure to identify any CS formation on these mutants upon 1 hr incubation at 42°C could not be due to cell death nor loss of heat shock response, since loss of viability of *wsc1Δ* and *bck1Δ* mutants was only 1 and 25% respectively (Additional file 2: Table S1), and both mutants readily accumulated trehalose in response to the thermal stress as wild type cells (Additional file 1: Figure S1). In addition, we failed to identify this structure on more than 20 independent analyzed *wsc1Δ* and *bck1Δ* mutant cells. Therefore, these results support an implication of CWI pathway in the formation of the cell surface CS. We also noticed that the YM of the unstressed *wsc1Δ* was comparable to the one determined on heat-shocked wild type cells (Additional file 8: Figure S7). Also, these unstressed *wsc1Δ* cells exhibited a chitin content twofold higher than the wild type cells (Additional file 9: Table S2), arguing in favor of a correlation between chitin content and stiffness of the cell wall. After exposure to 42°C for 1 h, the YM values and the chitin content in the *wsc1Δ* mutant were not significantly affected (Additional files 8 and 9: Figure S7 and Table S2).

## Discussion

The Atomic Force Microscopy (AFM) is nowadays the most powerful scanning microscopy tool used to visualize and to explore the dynamics of living cells at the nanometer resolution under physiological conditions. Being also a force machine, it allows force spectroscopy measurements of the cell mechanics [39]. Therefore, it is a superb method for investigating the biomechanical consequences of a heat shock on the yeast cell, with the eventual aim to correlate the putative biophysical changes observed using this methodology to the largely documented molecular and metabolic responses to heat shock [23]. In this study, we reported for the first time the formation of a circular structure (CS) that is induced upon exposure of yeast cell to 42°C. The high resolution AFM imaging clearly indicated that this singular feature takes its origin from a single point and propagates in concentric rings during the time of incubation at 42°C. In addition, this singular CS was observed in yeast cells immobilized by two different methods, which further supports the idea that the formation of this feature is a true morphological event induced by heat shock. The reason why Adya *et al*. [27] did not find this morphological event in their heat shock study by AFM could be explained by the immobilisation technique these authors used, which likely destroyed the integrity of the cell surface.

The discovery of only one singular CS per cell (although we could not preclude that another one was formed underside of the cell since this was not accessible to the AFM analysis), together with the close vicinity of this structure to a previous bud and with the fact that it appeared on about 40% of the heat shocked cells were indications that this amazing structure may be related to a failure in the budding emergence and/or in the budding process. This suggestion is supported by the inability of a mutant defective in *BNI1* encoding a formin protein that is needed for proper bud pattern formation to produce the CS in response to heat shock. The function of this protein is to assemble linear actin cables along the mother daughter axis and at the bud neck [40]. The polarization of the actin cytoskeleton is an essential process for cell expansion and budding in the yeast *S. cerevisiae*, and a defect in this process results in abnormal morphology characterized either by elongated buds or spherical buds [36]. Delley & Hall [41] have reported that a mild heat shock from 24 to 37°C induces a transient depolarization of the actin cytoskeleton that is accompanied by a transient depolarized distribution of the β-glucan synthase complex, composed of the catalytic subunits Fks1 or Fks2 and the regulatory subunit Rho1. They further showed that this depolarization of the actin cytoskeleton and β-glucan synthase was mediated by the plasma membrane protein Wsc1. Interestingly, we found that heat-induced formation of CS was abolished when latrunculin A, a toxin molecule known to disrupt actin cystokeleton [32], was added prior to the thermal stress, as well as in *wsc1Δ* mutant cells. In addition, the use of Calcofluor white staining method highlighted the presence of chitin rings at the vicinity of the CS outer ring. This finding is reminiscent of the presence of the chitin ring that delimitate the bud scars on the yeast cell surface [30]. Taken together, these results support the idea that the heat-induced formation of CS is a morphological consequence at the cell surface of a defective budding process due to perturbation of the actin cytoskeleton depolarization process.

It is known that the CWI pathway is activated under heat stress and although the cell surface mechanosensor Wsc1 is important in detecting this cell wall stress and to transmit the signal to the Pkc1 MAP kinase cascade [42], it is not the sole sensor implicated in the heat stress response [43]. Therefore, the finding that *bck1Δ* mutant cells, defective in the MAP kinase of the CWI pathway, could not produce this structure in response to the thermal stress indicates that the morphological process that leads to CS formation is indeed under the control of the CWI pathway.

The nanomechanical properties of yeast cells obtained from the AFM force volume curves showed that the heat stress caused a twofold increase in the Young’s Modulus values, indicating that the stiffness of the cell wall was increased (or its elasticity decreased). Interestingly, the measurement of cell wall β-glucans, mannans and chitin in yeast cells exposed to 42°C only showed an increase of approximately twofold of the chitin content. Moreover, the loss of *WSC1* resulted also in a twofold increase of both the chitin content and the Young’s Modulus. Taken together, these results suggest that the cell wall elasticity is mainly linked to the relative changes in the chitin content as described recently by Formosa *et* al. [44]. This result does not contradict our previous work showing that the cell wall elasticity was merely dependent on cross-linkages between chitin and β-glucans rather than on a particular cell wall component [6], since chitin content is in fact the most critical component that ensures strength of the cell wall through the covalent connection that it makes with the other cell wall components.

## Conclusions

The powerful technology AFM allowed identifying and precisely describing an unexpected morphological phenomenon occurring at the cell surface, which may explain physically how yeast cells are damaged by temperature stress and could eventually lead to cell death. Our results are also relevant in regards to the rough industrial growth conditions and processes which the yeast *S. cerevisiae* has to cope with, and which may cause comparable morphological defects at the cell surface.

## Materials and methods

### Yeast strains and growth conditions

Yeast strain BY4741 (MATa *his3Δ1 leu2Δ10 met15Δ0 ura3Δ0*) [45] and its isogenic deletion mutants *wsc1Δ*, *bck1Δ*, *chs1Δ*, *chs3Δ* and *bni1Δ* obtained from Open Biosystem (USA) were used in this study. Yeast cells were routinely cultivated at 30°C in a standard rich YEPD (Yeast Extract Peptone Dextrose) medium containing 10 g/l of yeast extract, 20 g/l of peptone and 20 g/l of dextrose. Heat shock experiments were carried out with exponentially growing cells (OD_600_ at 1–2 unit) by putting part of the yeast culture (10 mL in 50 mL Erlen flask) in a water bath set at 42°C during 1 h.

### Latrunculin A and nitrogen starvation experiments

Latrunculin [32] was added at 200 μM to exponentially growing cells cultivated at 30°C or just before transferring yeast culture cells at 42°C. For nitrogen starvation experiment, exponentially growing cells in YEPD (collected at OD_600_ at 1.0 unit) were washed 3 times in nitrogen-depleted medium (50 mM of phosphate without nitrogen, 2% of glucose, pH 6.2) and resuspended at OD_600_ of 1.0 unit in this medium for 72 h at 30°C before heat shock as described above.

### AFM sample preparation

Yeast cells were immobilized according to two different protocols. The first method consisted in filtering a small volume of yeast culture (1 to 5 mL) through a polycarbonate membrane pore sizes of 5 μm in order to trap cells into the micrometer size pores of the nylon filter (Merck Millipore, Darmstadt, Germany). After filtration, the filter was washed once with 4 mL of acetate buffer 20 mM, pH 5.5. In the second method, the cells were captured in microstructured polydimethylsiloxane (PDMS) stamps according to [28]. Briefly, 1 mL of the cell culture were washed quickly 3 times with 1 mL of AFM buffer (18 mM CH_3_COONa, 1 mM CaCl_2_ and 1 mM MnCl_2,_ pH 5.2), resuspended in 1 mL of the same buffer, and 100 μl of this cell suspension was deposited on a freshly oxygen activated microstructured PDMS stamp. The cells were allowed to stand for 15 min at room temperature and then forced to enter the microstructures of the stamp by convective/capillary assembly [28]. A typical example of cells immobilized in holes of a PDMS stamp is given in Additional file 10: Figure S8. To get statistical significance of the AFM data, about 10–12 cells have been analyzed from three independent experiments. In addition, three independent investigators performed AFM experiments. Each investigator has analyzed 10–12 cells.

### AFM imaging and Force spectroscopy experiments

AFM images of yeast cells trapped in polycarbonate membrane were recorded with a Nanowizard II form JPK (JPK Instruments, Berlin, Germany), in contact mode, using OTR4 (Olympus provided by Bruker) cantilevers. AFM experiments on yeasts immobilized on PDMS stamps were performed with a Nanowizard III form JPK (JPK Instruments, Berlin, Germany) in contact mode, Quantitative Imaging mode (QI) [46] and Force Volume mode (FV). The cantilevers used (OTR4 and MLCT) had a spring constant measured by the thermal noise method [47] ranging from 0.01 to 0.5 N/m. Cell wall elasticity was deduced from the Young’s modulus which was calculated from FV measurement using the Hertz model [48].

### Extraction of cell wall and determination of β-glucan and mannan polysaccharides

Yeast cells (about 50 mg dry mass or 10^9^ cells) were collected by centrifugation (5 min, 3000 g), washed once with 10 mL of cold sterilized water, and after a second centrifugation, cell pellet was resuspended in cold water. The cell walls (about 10 mg dry mass) obtained from control and heat shocked yeast cells were extracted according to the protocol described by Dallies *et al*. [49]. The content of β-glucans and mannans in the *S. cerevisiae* cell walls were determined by acid sulfuric hydrolysis method as described by François [50]. The released monosaccharides (glucose and mannose) were quantified by HPAEC-PAD on a Dionex-ICS 5000 system (Thermofisher Scientific, France). Separation was performed on a CarboPac PA10 analytical column (250 × 4 mm) with a guard column CarboPac PA10, by an isocratic elution of NaOH 18 mM at 25°C and a flow rate of 1 mL/min. Detection was performed by pulsed amperometric system equipped with a gold electrode.

### Analytical methods

Intracellular trehalose level was determined as previously described [51]. For accurate chitin determination in the yeast cell wall, an enzyme assay has been used as follows. Lyophilized cell walls (about 10 mg) were suspended in 200 μl of 50 mM potassium acetate buffer, pH 5.0 and boiled at 65°C for 5 min. After mixing and cooling to ambient temperature, the cell wall suspension was treated with 1U of chitinase from *Streptomyces griseus* (Sigma-Aldrich, France) for 24 h at 37°C. The *N*-acetyl glucosamine released by the chitinase action was then determined using a colorimetric method as described by Reissig *et al*. [52] and adapted for a micro method. Briefly, 125 μl of the enzymatic mixture was heated with 25 μl of 0.8 M potassium tetraborate pH 9.0 at 100°C for 8 minutes. After cooling at room temperature, 750 μl of Reissig reagent (10 g of 4-dimethylaminobenzaydedyde dissolve in 12.5 mL 10 N HCl and 87.5 mL of glacial acetic acid) diluted ten times in deionized water was added, and the tubes were incubated 40 minutes at 37°C. The absorbance was read at 585 nm. The chitin content was obtained from N-acetylglucosamine standard curve (from 0 to 100 μg/mL) made in the same condition.

### Miscellaneous methods

Calcofluor white treatment of yeast cells before and 1 hr after heat shock was carried out as following the procedure described in [53]. Cell viability was performed using methylene blue according to [54].

## Competing interests

We declare that we have no competing interest.

## Authors’ contributions

ED and JMF are the lead authors of the paper. FP and SL carried out the most of the AFM experiments. MS performed extraction and analysis of carbohydrates contents in yeast. CF worked on complementary AFM experiments. HMY participated in writing the paper and performed the biological part of the experiments. All authors read and approved the final manuscript.

## Supplementary Material

Additional file 1: Figure S1 The accumulation of trehalose is correlated with survival of cells under heat stress condition. Comparison of trehalose accumulation in the wild-type yeast BY4741 and the defective mutants *wsc1Δ* and *bck1Δ*. Control (full bar) and heat-shocked condition (hachured bar) are represented. Click here for file

Additional file 2: Table S1 Evaluation of viability by blue methylene test. The percentage of mortality was evaluated before and after heat shock with the defective mutants *wsc1* and *bck1*, and the wild-type yeast with or without nitrogen starvation during 72 h. Click here for file

Additional file 3: Figure S2 Young modulus increase with heat-shock. Distribution of Young modulus values calculate with 19 elasticity maps (n_curves_ = 19443) from individual yeasts unstressed **(A)**, in comparison with 15 elasticity maps (n_curves_ = 15307) from individual yeasts heat-shocked at 42°C **(B)**. YM medians were indicated on diagrams and calculated from fits in gauss model (red curves). **(C)** Statistic unpaired t test between averages and standard deviations calculated from young modulus values. The 3 asterisks shown significant differences between elasticity of unstressed yeasts (full bar) and heat-shock yeasts (hachured bar) at the P value < 0.0001. Click here for file

Additional file 4: Figure S3 The absence of F-Actin prevent the formation CS. AFM high resolution images of wild-type cells after heat shock in absence **(A)** or in presence of 200 μM Latrunculin A **(B)**. Cells incubated 1 hr at 30°C with 200 μM of Latrunculin A **(C)**. Click here for file

Additional file 5: Figure S4 The formation of CS require budding process. High-resolution deflection images of wild-type incubate 72 h at 30°C in nitrogen starvation, without **(A)** or with heat shock 1 hr at 42°C **(B)**. Click here for file

Additional file 6: Figure S5 The heat-induced formation of the cell surface circular structure is abolished in mutants defective in the budding process. High-resolution AFM deflection images of *bni1Δ***(A)**, *chs3Δ***(B)** and *chs1Δ***(C)** mutants after heat shock. Click here for file

Additional file 7: Figure S6 The CWI controls the stiffness of the cell wall and the formation of the cell surface circular structure in response to heat shock. High-resolution AFM deflection images of wild-type cell **(A)**, *wsc1Δ***(B)** and *bck1Δ***(C)** cell defective in the CWI pathway imaged after 1 hr of incubation at 42°C. Click here for file

Additional file 8: Figure S7 The stiffness of *wsc1Δ* unstressed was similar to wild-type yeast exposed at 42°C during 1 h. Distribution of Young modulus values calculate with 4 elasticity maps (n = 4096) from individual *wsc1Δ* yeasts unstressed. Click here for file

Additional file 9: Table S2 Chitin rate was similar in *wsc1Δ* mutant with or without heat-shock at 42°C. Carbohydrate composition of *wsc1Δ* mutant was determinated by acid hydrolysis and enzymatic method and expressed in μg/mg of cell wall dry mass. Click here for file

Additional file 10: Figure S8 Yeast immobilization on PDMS stamp. **(A)** AFM height image of a PDMS stamp containing some immobilized yeasts. The z range is 2.5 μm. **(B)** 3D projection associated to the height image. Click here for file
