# Supplementary material for: Uncovering by Atomic Force Microscopy of an original circular structure at the yeast cell surface in response to heat shock
Source: BMC Biol. 2014 Jan 27;12:6. doi: 10.1186/1741-7007-12-6 (PMC3925996; doi:10.1186/1741-7007-12-6)
Supplement: Additional file 1: Figure S1 — The accumulation of trehalose is correlated with survival of cells under heat stress condition. Comparison of trehalose accumulation in the wild-type yeast BY4741 and the defective mutants wsc1Δ and bck1Δ. Control (full bar) and heat-shocked condition (hachured bar) are represented. [file 1741-7007-12-6-S1.doc]

**Additional file 1: Figure S1. The accumulation of trehalose is correlated with survival of cells under heat stress condition.**Comparison of trehalose accumulation in the wild-type yeast BY4741and the defective mutants*wsc1Δ* and *bck1Δ*. Control (full bar) and heat-shocked condition (hachured bar) are represented.
