# Supplementary material for: Uncovering by Atomic Force Microscopy of an original circular structure at the yeast cell surface in response to heat shock
Source: BMC Biol. 2014 Jan 27;12:6. doi: 10.1186/1741-7007-12-6 (PMC3925996; doi:10.1186/1741-7007-12-6)
Supplement: Additional file 2: Table S1 — Evaluation of viability by blue methylene test. The percentage of mortality was evaluated before and after heat shock with the defective mutants wsc1 and bck1, and the wild-type yeast with or without nitrogen starvation during 72 h. [file 1741-7007-12-6-S2.doc]

|  | At 30°C | After HS 1h at 42°C |
| --- | --- | --- |
| wsc1 mutant | 1% | 1% |
| bck1 mutant | 1% | 25% |
| Wild-type | 0.2% | 1% |
| Wild-type (nitrogen starvation) | 10% | 25% |

**Additional file 2: Table S1. Evaluation of viability by blue methylene test.**The percentage of mortality was evaluated before and after heat shock with the defective mutants *wsc1* and *bck1*, and the wild-type yeast with or without nitrogen starvation during 72 h.
