# Supplementary material for: Uncovering by Atomic Force Microscopy of an original circular structure at the yeast cell surface in response to heat shock
Source: BMC Biol. 2014 Jan 27;12:6. doi: 10.1186/1741-7007-12-6 (PMC3925996; doi:10.1186/1741-7007-12-6)
Supplement: Additional file 3: Figure S2 — Young modulus increase with heat-shock. Distribution of Young modulus values calculate with 19 elasticity maps (ncurves = 19443) from individual yeasts unstressed (A), in comparison with 15 elasticity maps (ncurves = 15307) from individual yeasts heat-shocked at 42°C (B). YM medians were indicated on diagrams and calculated from fits in gauss model (red curves). (C) Statistic unpaired t test between averages and standard deviations calculated from young modulus values. The 3 asterisks shown significant differences between elasticity of unstressed yeasts (full bar) and heat-shock yeasts (hachured bar) at the P value < 0.0001. [file 1741-7007-12-6-S3.doc]

**Additional file 3:Figure S2. Young modulus increase with heat-shock.**Distribution of Young modulus values calculate with 19 elasticity maps (n_curves_ = 19443) from individual yeasts unstressed **(A),** in comparison with 15 elasticity maps (n_curves_ = 15307) from individual yeasts heat-shocked at 42°C **(B)**. YM medians were indicated on diagrams and calculated from fits in gauss model (red curves). **(C)**Statistic unpaired t test between averages and standard deviations calculated from young modulus values. The 3 asterisksshown significant differences between elasticity of unstressed yeasts (full bar) and heat-shock yeasts (hachured bar) at the P value < 0.0001.
