# Supplementary material for: Uncovering by Atomic Force Microscopy of an original circular structure at the yeast cell surface in response to heat shock
Source: BMC Biol. 2014 Jan 27;12:6. doi: 10.1186/1741-7007-12-6 (PMC3925996; doi:10.1186/1741-7007-12-6)
Supplement: Additional file 4: Figure S3 — The absence of F-Actin prevent the formation CS. AFM high resolution images of wild-type cells after heat shock in absence (A) or in presence of 200 μM Latrunculin A (B). Cells incubated 1 hr at 30°C with 200 μM of Latrunculin A (C). [file 1741-7007-12-6-S4.doc]

**Additional file 4: Figure S3.The absence of F-Actin prevent the formation CS.** AFM high resolution images of wild-type cells after heat shock in absence **(A)** or in presence of 200 µMLatrunculin A **(B)**. Cells incubated 1 hrat 30°C with 200 µM of Latrunculin A (**C)**.
