# Supplementary material for: Uncovering by Atomic Force Microscopy of an original circular structure at the yeast cell surface in response to heat shock
Source: BMC Biol. 2014 Jan 27;12:6. doi: 10.1186/1741-7007-12-6 (PMC3925996; doi:10.1186/1741-7007-12-6)
Supplement: Additional file 5: Figure S4 — The formation of CS require budding process. High-resolution deflection images of wild-type incubate 72 h at 30°C in nitrogen starvation, without (A) or with heat shock 1 hr at 42°C (B). [file 1741-7007-12-6-S5.doc]

**Additional file 5: Figure S4.The formation of CS require budding process.** High-resolution deflection images of wild-type incubate 72 h at 30°C in nitrogen starvation, without (**A)** or with heat shock 1 hr at 42°C (**B)**.
