# Supplementary material for: Uncovering by Atomic Force Microscopy of an original circular structure at the yeast cell surface in response to heat shock
Source: BMC Biol. 2014 Jan 27;12:6. doi: 10.1186/1741-7007-12-6 (PMC3925996; doi:10.1186/1741-7007-12-6)
Supplement: Additional file 6: Figure S5 — The heat-induced formation of the cell surface circular structure is abolished in mutants defective in the budding process. High-resolution AFM deflection images of bni1Δ(A), chs3Δ(B) and chs1Δ(C) mutants after heat shock. [file 1741-7007-12-6-S6.doc]

**Additional file 6:Figure S5. The heat-induced formation of the cell surface circular structure is abolished in mutants defective in the budding process.** High-resolution AFM deflection images of *bni1*Δ **(A)**, *chs3*Δ **(B)** and *chs1*Δ **(C**) mutants after heat shock.
