# Supplementary material for: Uncovering by Atomic Force Microscopy of an original circular structure at the yeast cell surface in response to heat shock
Source: BMC Biol. 2014 Jan 27;12:6. doi: 10.1186/1741-7007-12-6 (PMC3925996; doi:10.1186/1741-7007-12-6)
Supplement: Additional file 7: Figure S6 — The CWI controls the stiffness of the cell wall and the formation of the cell surface circular structure in response to heat shock. High-resolution AFM deflection images of wild-type cell (A), wsc1Δ(B) and bck1Δ(C) cell defective in the CWI pathway imaged after 1 hr of incubation at 42°C. [file 1741-7007-12-6-S7.doc]

**Additional file 7: Figure S6: The CWI controls the stiffness of the cell wall and the formation of the cell surface circular structure in response to heat shock.** High-resolution AFM deflection images of wild-type cell **(A)**, *wsc1*Δ **(B)** and *bck1*Δ **(C)** cell defective in the CWI pathway imaged after 1 hr of incubation at 42°C.
