# Supplementary material for: Uncovering by Atomic Force Microscopy of an original circular structure at the yeast cell surface in response to heat shock
Source: BMC Biol. 2014 Jan 27;12:6. doi: 10.1186/1741-7007-12-6 (PMC3925996; doi:10.1186/1741-7007-12-6)
Supplement: Additional file 8: Figure S7 — The stiffness of wsc1Δ unstressed was similar to wild-type yeast exposed at 42°C during 1 h. Distribution of Young modulus values calculate with 4 elasticity maps (n = 4096) from individual wsc1Δ yeasts unstressed. [file 1741-7007-12-6-S8.doc]

**Additional file 8: Figure S7.The stiffness of *wsc1*Δ unstressed was similar to wild-type yeast exposed at 42°C during 1 h.**Distribution of Young modulus values calculate with 4 elasticity maps (n = 4096) from individual *wsc1*Δ yeasts unstressed.
