# Supplementary material for: Uncovering by Atomic Force Microscopy of an original circular structure at the yeast cell surface in response to heat shock
Source: BMC Biol. 2014 Jan 27;12:6. doi: 10.1186/1741-7007-12-6 (PMC3925996; doi:10.1186/1741-7007-12-6)
Supplement: Additional file 9: Table S2 — Chitin rate was similar in wsc1Δ mutant with or without heat-shock at 42°C. Carbohydrate composition of wsc1Δ mutant was determinated by acid hydrolysis and enzymatic method and expressed in μg/mg of cell wall dry mass. [file 1741-7007-12-6-S9.doc]

**Additional file 9: Table S2. Chitin rate was similar in *wsc1*Δ mutant with or without heat-shock at 42°C.** Carbohydrate composition of*wsc1Δ*mutant was determinated by acid hydrolysis and enzymatic method and expressed in µg/mg of cell wall dry mass.
