# Supplementary material for: Uncovering by Atomic Force Microscopy of an original circular structure at the yeast cell surface in response to heat shock
Source: BMC Biol. 2014 Jan 27;12:6. doi: 10.1186/1741-7007-12-6 (PMC3925996; doi:10.1186/1741-7007-12-6)
Supplement: Additional file 10: Figure S8 — Yeast immobilization on PDMS stamp. (A) AFM height image of a PDMS stamp containing some immobilized yeasts. The z range is 2.5 μm. (B) 3D projection associated to the height image. [file 1741-7007-12-6-S10.doc]

**Additional file 10: Figure S8. Yeast immobilization on PDMS stamp.(A)** AFM height image of a PDMS stamp containing some immobilized yeasts. The z range is 2.5 µm.**(B)** 3D projection associated to the height image.
